# Vitamin D Knowledge and Awareness Is Associated with Physical Activity among Adults: A Cross-Sectional Survey

**DOI:** 10.3390/ijerph20021601

**Published:** 2023-01-16

**Authors:** Nasser M. Al-Daghri, Hanan A. Alfawaz, Nasiruddin Khan, Gamal M. Saadawy, Shaun Sabico

**Affiliations:** 1 Biochemistry Department, King Saud University, Riyadh 11451, Saudi Arabia; 2 Department of Food Science & Nutrition, College of Food & Agriculture Science, King Saud University, Riyadh 11495, Saudi Arabia; 3 Department of Food Science and Human Nutrition, College of Applied and Health Sciences, A’Sharqiyah University, Ibra 400, Oman

**Keywords:** vitamin D, knowledge, awareness, attitude, physical activity, Saudi Arabia

## Abstract

The relation between knowledge and awareness of vitamin D (VD) and physical activity in adults has not been well studied. The present cross-sectional study aims to demonstrate this relation among adults living in Saudi Arabia. A total of 774 adults participated and were stratified based on self-reports of whether they were physically active (PA group, N = 562) or not (non-PA, N = 212). The prevalence of VD awareness and its health effects were significantly higher in the PA group compared with their counterparts (97 vs. 93.4%; *p* = 0.02 and 92.3 vs. 81.6%; *p* < 0.001, respectively). Sunlight was the preferred overall source of VD among the PA group (91.1%) followed by food, supplements, and fortified foods. Sardine, salmon, oily fish (63.7%) and eggs (54.6%) were reported as good dietary sources. Other positive attitudes, represented by taking supplement or multivitamins (51.2%), high sun exposure (33.1%), and daily duration of exposure to sunlight (15–30 min; 53.4%) were higher in the PA than the non-PA group (*p* values < 0.05). The body parts exposed to the sun among the PA group were arms (67.1%), hands (64.1%), face and hands (62.5%), legs (58.5%), and face (53.2%), while sunscreen cream (47.2%) was their preferred sun-protection method. High levels of knowledge and positive attitudes were reported by the PA group compared with the non-PA group in regard to walking outdoors for sun exposure (80.6 vs. 62.7%; *p* < 0.001). Information about good dietary sources, the role of VD in human health, associated diseases, positive attitudes to sun exposure, and the use of supplements or multivitamins were among the determinants of VD knowledge and awareness. In conclusion, VD knowledge and awareness are positively associated with self-reported physical activity in adults. Further objective classifications of PA may strengthen the results of the present investigation.

## 1. Introduction

The prevalence of vitamin D (VD) deficiency is increasing worldwide and is reported to range from 20–90% in the Middle East, USA, and Europe [1,2]. A recent study conducted among South Asian countries (Bangladesh, India, Pakistan, Nepal, Bhutan, Maldives, Sri Lanka, and Afghanistan) reported a high prevalence of VD deficiency in the adult population [3]. Despite the plentiful sunshine, the situation is similar in Saudi Arabia with a high prevalence of VD deficiency among all ages and genders [4] Although a recent report from Saudi Arabia suggested improved serum levels of 25 (OH)D in the general population over time (2008–2017) [5], current evidence has also implicated VD deficiency in various disorders not previously documented in the country, including osteomalacia and different levels of intelligence in adolescents [6,7], as well as sarcopenia and premature biological aging in adults [8,9]. Such observations further fuel the need to avoid complacency when disseminating VD knowledge to the general community. 

Insufficient dietary intake including fish, milk products, and fortified foods, along with limited exposure to or avoidance of sunlight, traditional clothing, indoor activities, obesity, and unhealthy lifestyles are some of the common risk factors associated with VD deficiency in the Saudi population [10]. VD deficiency has been found to be related to increased risk of chronic non-skeletal diseases, including several communicable and non-communicable diseases such as diabetes, CVD, arthritis, breast cancer, and colon cancer [11,12]. In addition, VD is associated with homeostasis, and helps regulate the immune and endocrine systems [13].

Low levels of knowledge about VD and low supplement intake were reported to be causal factors for the high prevalence of VD deficiency among Saudi adults with pre-existing conditions and low PA [14,15]. In a recent study from UAE, higher overall VD knowledge was observed among adult Emiratis, but lower or medium levels of knowledge related to PA and awareness of VD supplements [16]. Similarly, a web-based Jordanian study reported lower levels of knowledge and practices regarding VD among university students [17]. In Malaysia, female office workers were observed to have a good VD knowledge (45%) but moderate attitude (76%) and practice (84%) in relation to sunlight exposure and dietary VD intake [18]. 

It is well established that regular outdoor PA [20–30 min] may help increase VD levels by as much as two to three times during the summer [19]. PA has been shown to affect hormonal changes which in turn can lead to changes in VD level [20]. Moreover, the level of VD increases during indoor muscle-building exercise because VD can be stored in muscles [21]. There is limited knowledge about VD and muscle function in the Saudi adult population. Adult Saudi females exhibited improved muscle strength after consuming oral VD supplementation [22] and similar results were obtained among the Asian Indian population [23].

Recent studies from different parts of Saudi Arabia have assessed awareness and knowledge about VD in relation to various lifestyle behaviors and risk factors among adult males and females [24,25,26,27]. However, none of these studies focused on whether VD awareness and knowledge could reflect an attitude towards PA, or provided any information in this context. Therefore, to fill this gap, the present cross-sectional study aims to determine the relation between VD knowledge, awareness, and PA among adults living in Saudi Arabia.

## 2. Materials and Methods

### 2.1. Study Design and Participants

The present cross-sectional study was conducted from 20 October to 8 December 2021. The survey aimed to investigate the knowledge, behaviors, and attitudes relating to VD and sun exposure among adult males and females (aged 18 years and above) living in Saudi Arabia. A consent form was completed by each participant prior to inclusion. A total of 774 individuals participated. Sample size was determined using the estimated population size (20,000 adults), accepted margin of error = 3.45%, level of confidence = 95%, and response distribution rate = 50%. The actual sample size = 774 was obtained using a Raisoft ^®^ sample-size calculator. This study was granted ethical approval in March 2018 from the Ethics Committee of the College of Science, Ref No: KSU-HE-21-379, King Saud University, Riyadh, Saudi Arabia. 

### 2.2. Questionnaire

A pilot test (N = 100 participants) was performed to ascertain the validity and reliability of the online survey questions included in the questionnaire. Appropriate changes were applied, based on feedback. The Cronbach’s coefficient reliability test range yielded 0.70–0.95 for each component of the questionnaire, with a Cronbach’s alpha of 0.90. No significant difference was observed in the reliability coefficient between males and females. The questionnaire included 24 questions, with both close-ended and multiple-choice options pertaining to VD knowledge, behaviors, attitudes, and sun exposure. For participants’ clarity and understanding, the cover letter was made available both in Arabic and English languages. The final version of the questionnaire was distributed through social media platforms (WhatsApp groups and Twitter accounts). One response per ID was permitted, to ensure no duplication of data. The questionnaire consisted of three parts, the first including baseline demographic data, social data (residence, marital status, education, family income, and smoking history), and anthropometric measurements (age and BMI). In the second part, questions included medical history and health data (if you may have VD deficiency will you take a test, taking VD supplements, VD supplement dose, motivation to take VD supplements. The final part consisted of questions regarding awareness and attitudes: VD sources, the role of VD in the body, sunlight exposure, use of sun blocker, length of day, season affecting VD synthesis).

### 2.3. Statistical Analysis

Data were analyzed using SPSS (version 22, IBM, Chicago, IL, USA). Continuous data are presented as mean ± standard deviation (SD) and categorical data as frequencies and percentages (%). All continuous variables were checked for normality using the Kolmogorov–Smirnov test, and independent T testing was performed between the PA status groups for age and BMI. VD knowledge, behavior, and attitude were analyzed on the basis of physical activity status using Chi-square and Fisher exact tests. Multinomial logistic regression was performed for odds ratio to physical activities status, for the VD knowledge categories of main source, dietary source and associated diseases. A *p* value < 0.05 was considered statistically significant.

## 3. Results

The demographic and anthropometric information of the subjects are shown in Table 1. The participants with PA exhibited a significantly lower BMI of 25.2 ± 5.8 kg/m^2^ (*p* = 0.002) compared with those without PA. The rest of the variables were not significantly different between groups. 

Table 2 shows the prevalence of medical and family histories according to PA status. The prevalence of diabetes mellitus (DM) (60.7 vs. 60.4%), dyslipidemia (23.7 vs. 19.3%), and arthritis (32.1 vs. 28.1%) was slightly high among families of PA vs. the non-PA group while hypertension (HTN) and heart disease were more prevalent among the non-PA group (60 vs. 61.3% and 26.5 vs. 30.2%, respectively).

VD source, attitude, and knowledge related to physical activities are shown in Table 3. The majority of the participants with PA were aware about VD and responded “yes” for questions such as “effect of VD on health is important”, to a greater extent than the non-PA group (97 vs. 93.4% and 92.3 vs. 81.6%, respectively). Around 91.1% of PA respondents reported sunlight as an overall source of VD, followed by food, supplements, and fortified foods (83.5, 81.7, and 61.6%, respectively). Good dietary sources of VD as reported by the majority of participants with PA were sardine, salmon, oily fish (63.7%), eggs (54.6%), and breast milk (52.5%). Moreover, the majority of the PA group responded positively to questions related to the role of VD including absorption of calcium and phosphorus, developing mineralization, aiding immune system function, and helping to strengthen muscles (60.7, 76.5, 69.6, 72.4%) compared with the non-PA group (55.7, 71.7, 57.1, and 55.2%, respectively).

A higher proportion (89%) of the PA group believed that “indoor working people were at high risk of VD deficiency” compared with the non-PA group (80.7%). Around 59.4% of the PA group responded yes to undergoing a test for VD, which was higher than the non-PA group (53.8%). About (78.8%) of the PA group were curious and interested to know more about VD. More than half of the PA group (51.2%) reported having used supplements or multivitamins at some point in their lives, with 24.9% (*p* = 0.029) taking a dose of approximately <1000 IU. When asked about their motivation for starting VD supplements, some (5.3%) stated that friends or family were a source of motivation, while the majority used supplements either due to VD deficiency (54.1%) or after recommendation from a doctor or health care professional (15.8%).

Positive responses to “are you getting enough sun exposure?” were higher in the PA group (33.1%) than the non-PA group (18.4%). Around 53.4% of the PA group participants reported their average length of daily stay in sun as 15–30 min, which was a higher rate than their counterparts (40.6%). When asked “which part of your body is generally exposed to sun?”, various positive responses were frequent in the PA group participants, including arms (67.1%), hands (64.1%), face and hands (62,5%), legs (58.5%), and face (53.2%). Moreover, the percentage of participants who completely covered their bodies was lower in the PA group (31%) than the non-PA group (33%). The responses from participants (PA vs. non-PA group) regarding the use of sun protection in summer and autumn ranged from “always” (22.4, 20.3%) to “never” (24.9, 39.6%, respectively). Among the PA group, around 47.2% used sunscreen cream, with others using hats (12.1%), or umbrellas (10.7%) as primary protection if exposed to the sun.

The majority of the participants in the PA group (80.6%) reported walking outdoors daily to obtain sufficient sun exposure, which was a higher rate than their non-PA counterparts (62.7%). Knowledgeable responses to the statement “season affect the amount of time in the sun required to synthesis adequate VD” was also high in the PA group (75.1%). Similarly, the frequency of positive responses from PA participants were higher (64.9%) when faced with the statements that “taking VD supplements, unless recommended by a physician, is wrong” and that it is “only necessary only in case of lack of exposure to sunlight” (58%), compared with the non-PA group (51.9, 54.7%, respectively). Table 4 represent the logistic regression analysis for PA and its association with other parameters (negative responses provide the reference).

## 4. Discussion

Knowledge, awareness, and positive attitudes among Saudi adults regarding VD and sun exposure were high, as specifically reflected among participants in the PA group. However, there is an urgent need for more information and awareness about sun exposure and related health effects, including skin care, VD supplements, and multivitamin use among the Saudi adult population. It is well established that physical inactivity is a modifiable risk factor for various diseases [28,29]. Our present study shows high prevalence of family history of disease among both the PA and non-PA group participants. 

Involvement in physical activity among the PA group with high prevalence of reported disease conditions in their family history may possibly reveal their precautionary actions, positive attitudes, knowledge, and awareness of PA and its association with various disease conditions. Their involvement in PA reveals their concern and their interest in follow a healthy lifestyle in order to overcome risk factors and avoid such diseases. In contrast, it is quite possible that non-involvement in physical activity could increase the risk factors for acquiring such family diseases among the non-PA group.

### 4.1. Awareness, Knowledge and Physical Activity among PA and Non-PA Group

In agreement with previous research, our present study demonstrated the frequency of knowledge among PA and non-PA groups regarding the main sources of VD such as “sunlight”. Overall knowledge was approximately equal compared with a previous local study (91.1% vs. 91%) [30] and lower than among British adults (91.1 vs. 99%) [31]. The respective probabilities of “having heard about VD” and agreeing that the “effect of VD on health is important” were 2.3-fold (95% CI: 1.09–4.68) and three-fold (95% CI1.17–7.68) higher in the PA compared with the non-PA group. Comparable with the observations reported by Babelghaith and colleagues [30], a higher proportion of PA group participants compared with non-PA responded correctly to statements about other sources of VD such as supplements (81.7 vs. 73%) and food sources including oily fish (63.7 vs. 45.8%) and egg (54.6 vs. 30.8%, respectively). Participants with PA also had a significantly higher probabilities of positive responses related to “good dietary sources of VD”, such as eggs at a probability of 1.6 (1.1–2.4), vegetables 1.6 (1.1–2.3), red meat 1.6 (1.0–2.5), and liver 1.8 (1.2–2.6), compared with those without PA.

As mentioned above, Malaysian females [18] showed good knowledge but moderate attitudes and practice regarding VD, while Emirati adults exhibited high awareness but low to medium knowledge about PA, calcium, and VD supplements [16]. The present results showed reasonable levels of knowledge about VD and its effect on bone health, main sources of VD such as sunlight, and other sources such as supplements. Our present study supports previous observations [18,32] showing a high prevalence of awareness about VD among all participants. 

It is well established that PA is associated with increased VD levels, higher bone mass, and reduced calcium excretion [33,34]. Moreover, in terms of its effect on immunity, VD supplementation is associated with reduced risk of upper respiratory tract infections in populations involved with PA [35], while increased immunity (increased secretory immunoglobulin A) has been demonstrated after VD supplement replacement in athletes [36]. Furthermore, the combined effect of VD supplements with PA helps chronic kidney disease (CKD) patients to improve muscle strength [37]. Other observations have included a positive linear association of PA with 25(OH)D levels in White subjects, with favorable synergistic effects on atherosclerotic cardiovascular disease in White and Black subjects [38], relief of depressive symptoms [39], and overall better quality of life [40]. 

There have been reports showing limited sun exposure and mixed attitudes towards the use of VD supplements, multivitamins, and fortified foods [41,42]. In fact, Saudi adults showed low levels of knowledge and consumption of multivitamins and VD supplements [14]. Similar to UK results [31], our present study demonstrated positive attitudes among 78% of PA group participants who expressed an urge to know more about VD. Also, a positive attitude was reported in the PA group, revealing that more than half (51.2%) were using either multivitamins or VD supplements and that majority use these as recommended by health professionals. 

### 4.2. Attitudes toward Sun Exposure and VD among PA and Non-PA Groups

Despite sufficient knowledge about sun exposure as a primary source of VD, a negative attitude towards sun exposure has been shown among Saudi females [25]. The high prevalence of VD deficiency in the Saudi Arabian population is due to limited exposure to direct sunlight. The main factors for this behavior include cultural and traditional practices (such as the abaya: a traditional garment that fully covers the skin), hot weather, and dark skin [6,7,14]. Therefore, it is essential that the Saudi citizens and residents rely on other sources of VD, such as food products enriched with VD and VD supplementation. Practices regarding sun exposure and VD that were reported in a Malaysian study included exposure of the face and hands among the majority of females [18]. In the Qassim region, the majority of subjects exposed their faces, arms, or legs to sunlight for <5 min (43.0%), or 5 to 15 min (30.4%). Only 17.2% of the participants used sunscreen when they were exposed to sunlight [27]. Methods employed to minimize sun exposure included covering the face, or the head, and wearing long sleeves (54.7, 41.3, and 46.5%, respectively) [43]. The use of sunscreen was more commonly reported in Arabian females in the general population [44,45], similar to the findings for Canadian females [46]. 

In relation to UV and health effects, previous Saudi studies have reported awareness among participants of skin cancer, related death, and skin, hence encouraging them to adopt different ways of using primary sun protection and sunscreen [43]. Our study corroborates this finding, showing sufficient positive attitudes towards sun exposure and protection in the PA group. The probability of positive responses in relation to obtaining sufficient sun exposure was twice as high at 2.2 (1.5–3.2) among the PA group compared with their non-PA counterparts, which partially supports Kuwaiti findings that also showed a positive attitude towards sunlight exposure [42]. In addition, the practice of walking outdoors (80.6%) to attain sufficient sun exposure indicates their healthy practices and positive attitudes, revealing their capacity to translate their knowledge about VD into daily lifestyle behaviors. In our present study, individuals with PA were 3.6 times more likely to walk in sunlight than those without PA. This is encouraging, since in Saudi Arabia the fortification of food with VD has not been fully implemented [47], despite national and regional recommendations [48,49]. 

The authors acknowledge several limitations to the current study. First, the classification of participants into PA and non-PA groups was based only on participant responses (yes or no). This approach is subject to known biases affecting self-reported measures, including social desirability and acquiescence [50], which may also explain why there were more PA participants than non-PA. This, however, was somehow controlled with use participant anonymity. Second, the study had more female than male respondents, which could bias our findings about sun exposure, sunscreen use, and protection against the sun. Moreover, our results might be subject to recall bias due to the online self-reporting method used when collecting questionnaire responses. Further research should investigate the same section of the population, using a more detailed questionnaire including questions about types and dosage of different VD supplements and multivitamins, sunscreen, skin health, and barriers to sun exposure specifically in females.

## 5. Conclusions

In conclusion, high levels of knowledge and awareness as well as positive attitudes towards VD and sun exposure may reflect involvement in PA as a favorable health behavior among Saudi adults. The determinants of VD knowledge and awareness included knowledge about good dietary sources of VD and diseases associated with VD deficiency, as well as positive attitudes towards VD relating to sun exposure and use of VD supplements and multivitamins, all of which were associated with PA. The presence of low and mixed levels of awareness and knowledge among Saudi adults about the use of sun protection requires the provision of more information via trustworthy sources including health professionals. The present study is a reminder to the Saudi health authorities and other relevant organizations that the majority of the Saudi adult population still needs to be educated about the favorable effects of VD supplementation and the consequences of VD deficiency. For this purpose, the Saudi health authorities must ascertain and encourage the use of VD supplements and multivitamins among all age groups.

## Figures and Tables

**Table 1:** Demographic and anthropometric information of the subjects.

Parameters	Physical Activities		*p* Value
	Yes	No	
Sex (M/F)	562 (180/382)	212 (59/153)	
Age	30.7 ± 12.6	31.0 ± 12.1	0.77
BMI (kg/m^2^)	25.2 ± 5.8	26.7 ± 6.6	0.002
Residence Status			
City	524 (93.2)	197 (92.9)	0.49
Village	38 (6.8)	15 (7.1)	
Marital Status			
Single	276 (49.1)	105 (49.5)	
Married	259 (46.1)	93 (43.9)	0.62
Widowed	13 (2.3)	5 (2.4)	
Divorced	14 (2.5)	9 (4.2)	
Education Status			0.71
University	306 (54.4)	112 (52.8)	
Postgraduate	87 (15.5)	38 (17.9)	
Secondary	135 (24.0)	45 (21.2)	
Primary	29 (5.2)	14 (6.6)	
Read and write	5 (0.9)	3 (1.4)	
Income Status			0.27
<5000 SAR	99 (17.6)	44 (20.8)	
5001–15,000 SAR	252 (44.8)	101 (47.6)	
>15,000 SAR	211 (37.5)	67 (31.6)	
Smoking Status			0.52
Frequent	31 (5.5)	12 (5.7)	
Occasional	25 (4.4)	15 (7.1)	
Ex-smoker	24 (4.3)	8 (3.8)	
Never smoked	482 (85.8)	177 (83.5)	

Note: Data presented as N (%). *p* value significant at <0.05 using Chi-square and Fisher exact tests.

**Table 2:** Frequency of family history related to PA.

Parameters	Physically Active	
	Yes	No
Sex (M/F)	562 (180/382)	212 (59/153)
Family History		
DM	341 (60.7)	128 (60.4)
HTN	337 (60.0)	130 (61.3)
Heart disease	149 (26.5)	64 (30.2)
Dyslipidemia	133 (23.7)	41 (19.3)
Cancer	84 (14.9)	36 (17.0)
Osteoporosis	109 (19.4)	50 (23.6)
Arthritis	68 (32.1)	158 (28.1)
Bone fracture	23 (10.8)	52 (9.3)
Medical History		
DM	51 (9.1)	21 (9.9)
HTN	48 (8.5)	23 (10.8)
Heart disease	15 (2.7)	10 (4.7)
Dyslipidemia	29 (5.2)	16 (7.5)
Cancer	4 (0.7)	3 (1.4)
Osteoporosis	15 (2.7)	11 (5.2)
Arthritis	30 (5.3)	18 (8.5)
Bone fracture	10 (1.8)	8 (3.8)

Note: Data presented as N (%). *p* value significant at 0.05 and 0.01 level using Chi-square and Fisher exact tests.

**Table 3:** Vitamin D sources and knowledge according to physical activity status.

Parameters	Physically Active		*p* Value
	Yes	No	
Gender (M/F)	562 (180/382)	212 (59/153)	
Have you ever heard about VD?			0.02
Yes	545 (97.0)	198 (93.4)	
No	17 (3.0)	14 (6.6)	
VD belongs to			0.15
Fat-soluble vitamins	157 (27.9)	45 (21.2)	
Soluble vitamins, all solutions	42 (7.5)	21 (9.9)	
Water-soluble vitamins	72 (12.8)	23 (10.8)	
Don’t know	291 (51.8)	123 (58.0)	
Effect of VD on health is important			<0.001
Yes	519 (92.3)	173 (81.6)	
No	9 (1.6)	9 (4.2)	
Don’t know	34 (6.0)	30 (14.2)	
Overall source of VD			
Food	469 (83.5)	165 (77.8)	0.17
Supplements	459 (81.7)	157 (74.1)	0.04
Exercise	142 (25.3)	34 (16.0)	0.01
Air	92 (16.4)	22 (10.4)	0.11
Sunlight	512 (91.1)	186 (87.7)	0.028
Fortified food	346 (61.6)	129 (60.8)	0.49
Water	122 (21.7)	34 (16.0)	0.22
Dietary source sufficient for VD level			0.037
Yes	159 (28.3)	51 (24.1)	
No	293 (52.1)	115 (54.2)	
Don’t know	110 (19.6)	46 (21.7)	
Good dietary sources of VD			
Fruits	262 (46.6)	85 (40.1)	0.10
Sardine, salmon, oily fish	358 (63.7)	124 (58.5)	0.41
Cow’s milk	212 (37.7)	67 (31.6)	0.26
Eggs	307 (54.6)	93 (43.9)	0.03
Chicken	116 (20.6)	31 (14.6)	0.15
Vegetables	257 (45.7)	76 (35.8)	0.04
Infant or toddler formula	235 (41.8)	92 (43.4)	0.49
Red meat	129 (23.0)	36 (17.0)	0.08
Bread	53 (9.4)	20 (9.4)	0.78
Breast milk	295 (52.5)	95 (44.8)	0.14
Fortified cow’s milk	212 (37.7)	82 (38.7)	0.54
Liver	208 (37.0)	61 (28.8)	0.010
Others	93 (16.5)	39 (18.4)	0.24
Indoor workers at high risk of VD deficiency			0.001
Yes	500 (89.0)	171 (80.7)	
No	23 (4.1)	8 (3.8)	
Don’t know	39 (6.9)	33 (15.6)	
Role of VD in body			
Absorption of calcium and phosphorus	341 (60.7)	118 (55.7)	0.45
Antioxidant	212 (37.7)	77 (36.3)	0.20
Developing mineralization	430 (76.5)	152 (71.7)	<0.001
Aiding with immune system function	391 (69.6)	121 (57.1)	0.001
Helping to strengthen muscles	407 (72.4)	117 (55.2)	<0.001
Needed for blood clotting	140 (24.9)	42 (19.8)	0.07
Will undergo a test for VD			0.28
Yes	334 (59.4)	114 (53.8)	
No	98 (17.4)	38 (17.9)	
Don’t know	130 (23.1)	60 (28.3)	
Taking VD supplements, unless recommended by physician, is wrong			0.001
Yes	365 (64.9)	110 (51.9)	
No	107 (19.0)	46 (21.7)	
Don’t know	90 (16.0)	56 (26.4)	
Are you getting enough sun exposure? (awareness)			<0.001
Yes	186 (33.1)	39 (18.4)	
No	340 (60.5)	153 (72.2)	
Don’t know	36 (6.4)	20 (9.4)	
Average length of daily sun exposure			<0.001
<15 min	186 (33.1)	67 (31.6)	
15–30 min	300 (53.4)	86 (40.6)	
30–60 min	36 (6.4)	23 (10.8)	
>60 min	9 (1.6)	6 (2.8)	
None	31 (5.5)	30 (14.2)	
Parts of the body exposed to the sun			
Face	299 (53.2)	104 (49.1)	0.17
Hands	360 (64.1)	135 (63.7)	0.52
Face and hands	351 (62.5)	129 (60.8)	0.39
Both arms	377 (67.1)	120 (56.6)	0.006
Both legs	329 (58.5)	106 (50.0)	0.011
Completely covered	174 (31.0)	70 (33.0)	0.31
Walk outdoor daily for sufficient exposure			<0.001
Yes	453 (80.6)	133 (62.7)	
No	109 (19.4)	79 (37.3)	
Taking supplement is only necessary in cases of lack of exposure to sunlight			0.42
Yes	326 (58.0)	116 (54.7)	
No	147 (26.2)	54 (25.5)	
Don’t know	89 (15.8)	42 (19.8)	
Sun protection in summer and autumn			0.001
Always	126 (22.4)	43 (20.3)	
Never	140 (24.9)	84 (39.6)	
Often	104 (18.5)	24 (11.3)	
Rarely	83 (14.8)	28 (13.2)	
Sometimes	109 (19.4)	33 (15.6)	
If I am exposed to the sun, I apply primary sun protection			0.016
Hat	68 (12.1)	23 (10.8)	
Sunscreen cream	265 (47.2)	83 (39.2)	
Umbrella	60 (10.7)	17 (8.0)	
Unscreened	95 (16.9)	42 (19.8)	
Other	74 (13.2)	47 (22.2)	
The season affects the amount of time in the sun required to synthesis adequate VD			<0.001
Yes	422 (75.1)	128 (60.4)	
No	53 (9.4)	31 (14.6)	
Don’t know	87 (15.5)	53 (25.0)	
Are you interested to know more about VD?			0.002
Yes	443 (78.8)	146 (68.9)	
No	70 (12.5)	29 (13.7)	
Don’t know	49 (8.7)	37 (17.5)	
Do you walk in sunlight?			<0.001
Yes	105 (18.7)	17 (8.0)	
Sometimes	191 (34.0)	38 (17.9)	
No	266 (47.3)	157 (74.1)	
Disease listed associated with low VD			
Breast cancer	52 (9.3)	18 (8.5)	0.43
Skin cancer	112 (19.9)	31 (14.6)	0.05
Type1 DM	118 (21.0)	31 (14.6)	0.03
Inflammatory bowel disease	90 (16.0)	25 (11.8)	0.08
Multiple sclerosis	130 (23.1)	36 (17.0)	0.04
Rheumatoid arthritis	215 (38.3)	84 (39.6)	0.39
Depression	356 (63.3)	125 (59.0)	0.15
Renal disease	94 (16.7)	45 (21.2)	0.09
Bone disease	366 (65.1)	121 (57.1)	0.024
Gallstones	68 (12.1)	32 (15.1)	0.16
Heart disease	108 (19.2)	25 (11.8)	0.008
Rickets	284 (50.5)	83 (39.2)	0.003
Osteoporosis	374 (66.5)	138 (65.1)	0.38
Ever taken supplements or multivitamins containing VD (Yes)	288 (51.2)	90 (42.5)	<0.001
VD dose			0.029
<1000 IU	140 (24.9)	34 (16.0)	
>1000 IU	124 (22.1)	50 (23.6)	
Don’t know	298 (53.0)	128 (60.4)	
Motivation for starting VD supplementation			0.016
Friends or family members	30 (5.3)	21 (9.9)	
Doctor or health care professional	89 (15.8)	33 (15.6)	
VD deficiency	304 (54.1)	105 (49.5)	
Read about VD on internet, social media	82 (14.6)	27 (12.7)	
School or university	37 (6.6)	24 (11.3)	
Seminar or workshop related to VD	20 (3.6)	2 (0.9)	

Note: Data presented as N (%). *p* value significant at 0.05 and 0.01 level using Chi-square and Fisher exact tests.

**Table 4:** Logistic regression analysis for PA with other parameters (negative response as the reference).

Parameters	Odds Ratio (95% CI)	*p* Value	Adjusted BMI *p* Value
Have you ever heard about VD (Yes)	2.27 (1.09–4.68)	0.027	0.19
Effect of VD on health is important (Yes)	3.0 (1.17–7.68)	0.022	0.08
Overall source of VD (yes)			
Supplements	1.36 (0.83–2.22)	0.22	0.21
Exercise	1.92 (1.24–2.95)	0.003	0.005
Air	1.69 (1.02–2.81)	0.044	0.04
Sunlight	0.63 (0.23–1.68)	0.35	0.33
Fortified food	1.19 (0.79–1.77)	0.39	0.41
Water	1.46 (0.93–2.26)	0.09	0.10
Dietary source sufficient for VD level (Yes)	1.22 (0.84–1.79)	0.30	0.32
Good dietary source of VD (yes)			
Fruits	1.16 (0.79–1.67)	0.44	0.49
Sardine, salmon, oily fish	1.25 (0.82–1.89)	0.30	0.27
Cow’s milk	1.27 (0.86–1.88)	0.22	0.24
Eggs	1.61 (1.09–2.37)	0.016	0.006
Chicken	1.57 (0.99–2.48)	0.052	0.04
Vegetables	1.57 (1.09–2.27)	0.016	0.01
Infant or toddler formula	0.83 (0.56–1.22)	0.35	0.31
Red meat	1.62 (1.05–2.52)	0.03	0.046
Bread	1.05 (0.60–1.83)	0.87	0.85
Breast milk	1.28 (0.87–1.89)	0.22	0.12
Fortified cow’s milk	0.86 (0.58–1.26)	0.43	0.57
Liver	1.75 (1.20–2.57)	0.004	0.002
Indoor workers are at high risk of VD deficiency (yes)	1.93 (1.26–2.97)	0.003	0.017
Role of VD in body			
Absorption of calcium and phosphorus	1.22 (0.77–1.93)	0.39	0.40
Antioxidant	0.86 (0.57–1.30)	0.47	0.48
Developing mineralization	0.59 (0.32–1.08)	0.09	0.06
Aiding immune system function	1.35 (0.86–2.13)	0.20	0.34
Helping to strengthen muscles	1.56 (0.99–2.45)	0.052	0.06
Needed for blood clotting	1.12 (0.72–1.74)	0.63	0.56
Will undergo a test for VD (Yes)	1.14 (0.74–1.75)	0.56	0.72
Taking VD supplements, unless recommended by a physician, is wrong			
Yes	1.43 (0.95–2.14)	0.09	0.17
Are you getting enough sun exposure? (Yes)	2.19 (1.49–3.24)	<0.001	<0.001
Average length of daily sun exposure			
<15 min	2.69 (1.51–4.77)	0.001	0.002
15–30 min	3.38 (1.94–5.89)	<0.001	<0.001
30–60 min	1.52 (0.73–3.13)	0.26	0.40
>60 min	1.45 (0.46–4.58)	0.53	0.45
None	Ref		
Which parts of your body are exposed to the sun? (Yes)			
Face	1.18 (0.86–1.62)	0.30	0.54
Hands	1.0 (0.72–1.40)	0.98	0.53
Face and hands	1.06 (0.076–1.46)	0.74	0.80
Both arms	1.55 (1.12–2.14)	0.009	0.030
Both legs	1.52 (1.07–2.13)	0.018	0.07
Completely covered	0.90 (0.64–1.27)	0.56	0.46
Walk outdoors daily for sufficient exposure (Yes)	2.47 (1.74–3.50)	<0.001	<0.001
Taking supplements is only necessary if lacking exposure to sunlight (Yes)	1.03 (0.71–1.51)	0.87	0.80
Sun protection in summer and autumn			
Always	Ref:		
Never	0.57 (0.37–0.88)	0.012	0.07
Often	1.48 (0.84–2.59)	0.17	0.08
Rarely	1.01 (0.58–1.76)	0.96	0.72
Sometimes	1.13 (0.67–1.89)	0.65	0.50
If I am exposed to the sun, I have to apply primary sun protection			
Hat	1.31 (0.72–2.37)	0.38	0.36
Sunscreen cream	1.41 (0.91–2.20)	0.12	0.17
Umbrella	1.56 (0.82–2.98)	0.18	0.15
Other	0.70 (0.42–1.16)	0.17	0.41
Unscreened	Ref:		
Season affects amount of time to synthesize adequate VD (Yes)	1.93 (1.20–3.13)	0.008	0.017
Are you interested to know more about VD (Yes)	1.26 (0.78–2.01)	0.34	0.33
Do you walk in sunlight?			
Yes	3.65 (2.11–6.32)	<0.001	<0.001
Sometimes	2.97 (1.99–4.43)	<0.001	<0.001
No	Ref:		
Diseases listed are associated with low VD (Yes)			
Breast cancer	1.10 (0.63–1.93)	0.74	0.71
Skin cancer	1.45 (0.94–2.24)	0.09	0.07
Type1 DM	1.55 (1.01–2.39)	0.046	0.08
Inflammatory bowel disease	1.43 (0.89–2.29)	0.14	0.15
Multiple sclerosis	1.47 (0.98–2.21)	0.064	0.06
Rheumatoid arthritis	0.94 (0.68–1.31)	0.73	0.91
Depression	1.20 (0.87–1.66)	0.26	0.36
Renal disease	0.75 (0.50–1.11)	0.15	0.12
Bone disease	1.40 (1.01–1.94)	0.039	0.04
Gallstones	0.77 (0.49–1.22)	0.27	0.26
Heart disease	1.78 (1.12–2.83)	0.016	0.02
Rickets	1.59 (1.15–2.19)	0.005	0.001
Osteoporosis	1.07 (0.77–1.49)	0.70	0.88
Ever taken supplements or multivitamins containing VD (Yes)	1.21 (0.86–1.69)	0.28	0.27
VD dose of supplement			
<1000 IU	Ref:		
>1000 IU	0.60 (0.37–0.99)	0.046	0.06

Note: Data presented as odds ratio (95% CI). *p* value significant at 0.05 and 0.01 level, adjusted and unadjusted model. Dependent parameters: Physical activity (yes = 1 and No = 0). Independent parameters: negative response taken as reference.

## Data Availability

The data presented in this study are available on request from the corresponding author. The data are not publicly available due to privacy protection.
